# BegiBrainTool: An Open‐Source Toolbox for Evaluation of Visual Function in Alzheimer's Disease

**DOI:** 10.1002/alz70856_100749

**Published:** 2025-12-25

**Authors:** Unai Sainz‐Lugarezaresti, Asier Erramuzpe, Iñigo Gabilondo

**Affiliations:** ^1^ Biobizkaia HRI, Barakaldo, Bizkaia, Spain; ^2^ University of the Basque Country, Leioa, Bizkaia, Spain; ^3^ Ikerbasque, the Basque Foundation for Science, Bilbao, Bizkaia, Spain

## Abstract

**Background:**

The aging population in Europe has led to an expected increase in neurodegenerative diseases such as Alzheimer's. Neurologists are demanding customized tools to better address this growing challenge. Currently, there is no comprehensive toolbox for evaluating visual capacities linked to early detection of these disorders. BegiBrainTool aims to fill this gap by providing a computerized modular battery of visual tests designed to assess spatial and dynamic vision, as well as autonomic visual responses, making it a valuable tool for clinical and research applications.

**Method:**

BegiBrainTool consists of three interactive modules, all implemented in PsychoPy (Peirce et al., 2019). The first module, focused on spatial vision, evaluates elementary and semantic visual processing by employing stimuli that vary in spatial frequency, contrast or luminosity, and color thresholds. The second module examines dynamic vision and eye‐tracking performance through tasks that include moving stimuli, visual search paradigms, and flicker fusion threshold tests. This module is enhanced with integrated eye‐tracking, which enables precise measurement of gaze behavior and pupilometry. Finally, the autonomic response module measures unconscious reactions to elemental and emotional visual stimuli, utilizing metrics such as pupillary response, heart rate variability, and galvanic skin response.

The modular structure of BegiBrainTool ensures that the tests are user‐friendly and customizable, facilitating adaptability to diverse clinical and research settings.

**Result:**

This project is currently ongoing. Preliminary efforts focus on validating the toolbox components and establishing robust protocols for data collection. Anticipated outcomes include a validated open‐source tool ready for clinical deployment.

**Conclusion:**

BegiBrainTool introduces a novel and interdisciplinary approach to visual assessment for neurodegenerative disorders. Using modular, computerized tools and advanced biometrics, it offers a powerful platform for patient monitoring and longitudinal analysis. BegiBrainTool has the potential to support neurologists in managing the increasing wave of neurodegenerative cases, while simultaneously contributing to research on the visual and autonomic markers of Alzheimer's disease.

